# Electromechanical Impedance Response in CMUT-Based Gas Sensors Exposed to Volatile Organic Compounds

**DOI:** 10.3390/s26082505

**Published:** 2026-04-18

**Authors:** Dovydas Barauskas, Mindaugas Dzikaras, Darius Viržonis, Donatas Pelenis

**Affiliations:** 1 Faculty of Technological Sciences, Panevėžio Kolegija/State Higher Education Institution, Laisvės Sq. 23, LT-35200 Panevėžys, Lithuania; 2 Faculty of Mechanics, Vilnius Gediminas Technical University, Saulėtekio al. 11, LT-10223 Vilnius, Lithuania

**Keywords:** cmut, gas sensors, volatile organic compounds, mass loading effect, resonance frequency, physisorption, adsorption-based sensing

## Abstract

A capacitive micromachined ultrasonic transducer (CMUT) was engineered and functionalized with either zeolitic imidazolate framework-8 (ZIF-8) dispersed in an AZ1512HS photoresist matrix or with graphene oxide (GOx) to operate as a gravimetric sensor for organic vapors. The sensor response was investigated under controlled humidity conditions during pulsed exposure to acetone, ethyl methyl ketone, isopropanol, kerosene, and diesel vapors. The impedance of the device was monitored by observing and tracking the resonance frequency shift as well as the resistance maximum shift, giving us the possibility to track two response parameters simultaneously. Different combinations of shifts in the sensor resonance frequency and the resistance maximum values were observed for the ZIF-8 functionalized device when exposed to the selected vapors, ranging from 12.4 kHz for ethyl methyl ketone to 2.4 kHz for diesel, and from 580 Ω for acetone to 20 Ω for isopropanol. Sensors functionalized with GOx did not demonstrate any significant response to either ethyl methyl ketone or isopropanol in the frequency domain. GOx-functionalized sensors were used for relative humidity monitoring in test gases. Besides the conventional response of the produced gravimetric sensing system, we also observed a strong relationship between the humidity of the gas mixture and the strength of the interaction of target gases with the functional film of the sensor. The results highlight the multidimensional nature of the sensor response and demonstrate how humidity influences the interaction between vapor molecules and the functional coating. This paper focuses on the characterization of the coupled behavior of resonance frequency and resistance shifts under controlled operating conditions. The presented experimental setup provides a basis for future concentration-dependent investigations and functional material comparisons in CMUT-based gravimetric sensing systems and provides a necessary foundation for accurate interpretation of future concentration-resolved measurements.

## 1. Introduction

Gas sensing technologies play a crucial role in a wide range of applications, from industrial safety and environmental monitoring to healthcare diagnostics and indoor air quality management. Over the years, various gas sensing methods have been developed, each with its own strengths and limitations. As a recent trend replacing traditional electrochemical sensors, the Non-Dispersive Infrared (NDIR) technique is worth mentioning [1]. These sensors detect gases by measuring the absorption of infrared radiation at specific wavelengths characteristic of molecular bonds. They are widely used due to their selectivity and stability, particularly for gases such as CO_2_ and CH_4_, and represent a state-of-the-art optical sensing approach. The popularity of these sensors is due to the ability of CO_2_, CH_4_ and other molecules to absorb specific wavelengths of infrared light. Still, the selectivity of the NDIR technique is limited in some cases as the IR absorption bands of different molecules in a gas mixture often overlap. Also, they are inherently not suitable for detecting gases that do not absorb IR, such as monatomic and homonuclear. Complex organic compound gas molecules with convoluted IR spectra can also be challenging or impossible to differentiate. Additionally, the present designs of NDIR sensors are comparatively bulky and relatively expensive. This limits their integration into compact and portable devices [1]. Fourier-Transform Infrared (FTIR) spectroscopy is another infrared technique that offers high sensitivity, high selectivity (thus solving the issue of overlapping absorption bands), and multi-gas detection capabilities. It is commonly employed in industrial and environmental applications where high reliability and selectivity of gas detection are essential. While FTIR provides detailed spectral information, the equipment is generally expensive, bulkier compared to MEMS-based sensing solutions and less suitable for applications with limited availability of power, specifically for miniaturized, portable, or Internet of Things (IoT) use [2].

Chemiresistive gas sensors are another class of sensors, although sometimes they are considered as a type of electrochemical sensor. They operate by detecting changes in electrical resistance upon exposure to target gases. They have gained popularity in the past due to their simplicity and low cost. These sensors are often utilized in automotive, industrial, and household applications for detecting gases like CO, NO_2_, and VOCs (volatile organic compounds). Although chemiresistive sensors can be highly sensitive, their performance is susceptible to environmental factors such as temperature and humidity, which can affect accuracy and stability over time [3]. Also, achieving high performance of chemiresistive sensors is related to significant energy demands, thus reducing the autonomy of wireless or individual sensors.

Electrochemical sensing techniques, such as metal oxide semiconductor (MOS) gas sensors [4], offer various trade-offs in terms of sensitivity, selectivity, response time, and cost-effectiveness.

Reliable detection of volatile organic compounds is essential for air quality monitoring and human health. VOCs such as benzene, toluene, xylene, formaldehyde, acetone, and isopropanol are commonly encountered in industrial and indoor environments. Many of these compounds pose health risks: benzene is carcinogenic, formaldehyde is toxic and irritant even at low concentrations, and organic solvents such as acetone and isopropanol can affect the central nervous system during prolonged exposure. However, existing gas sensing technologies face challenges in balancing sensitivity, selectivity, and stability while maintaining compact size and low power consumption. The advantages and disadvantages of known traditional gas sensing technologies are summarized in Table 1, which highlights the strengths and limitations associated with each method.

Given the limitations of traditional gas sensors such as amperometric and potentiometric sensors, which operate by measuring current or voltage changes resulting from redox reactions between target gases and an electrolyte, there is a need for research into new sensing platforms that can address these challenges. Emerging applications demand smaller, more sensitive, and cost-efficient gas sensors, leading to increased interest in alternative platforms such as microelectromechanical systems (MEMS). MEMS technology enables the fabrication of highly compact and integrated devices with low energy consumption, making it an ideal choice for portable and wearable sensing applications. Various MEMS gas sensors have been employed for gas detection [12,13,14]. The sensors tend to use film bulk acoustic resonator (FBAR), surface acoustic wave (SAW), piezoelectric micromachined ultrasonic transducer (PMUT) and cantilever designs and are able to detect CH_4_, NH_3_, NO_2_, CO and other gases. Of particular interest are the VOCs, since they are more complex than inorganic molecules in their size and chemical bond distribution and much harder to differentiate in a gravimetric context. Beardslee et al. have demonstrated an intricate cantilever type device, able to detect m-xylene, toluene and benzene when coated with poly(isobutylene) [15]. Other VOC-detecting MEMS sensors have also been reported [16,17,18,19], demonstrating the advancing field and desire to produce affordable and reliable MEMS sensors. A functionalized electrostatics-based MEMS device has also been demonstrated to be sensitive to isopropanol vapor when coated with a polyaniline and ZnO mixture, albeit the device design was elaborate and complex [20].

The use of electrical impedance spectroscopy, based on tracking changes in the input impedance of the MEMS device, has also been reported for metal oxide-based MEMS gas sensors [21]. However, these gas sensors are degradation-prone since chemical reactions take place on their surface due to heating.

Surface-functionalized capacitive micromachined ultrasonic transducers (CMUTs), a class of MEMS devices, are particularly promising for gas sensing due to their ability to detect minute mass changes on their surface. A surface-functionalized CMUT refers to a device in which a thin gas-sensitive layer is deposited on top of the vibrating membrane, enabling interaction with target molecules while preserving the electromechanical operation of the transducer. CMUTs operate by applying an AC voltage superimposed on a DC bias, which induces electrostatic forces and causes the vibration of suspended membranes. The resonance frequency of the membrane is sensitive to changes in effective mass and mechanical properties, and the most dominant factor determining it is the membrane diameter, followed by thickness and material properties. In our research the surface-functionalized CMUTs operate by measuring shifts in resonance frequency caused by the adsorption of gas molecules onto their surface, which makes them highly sensitive to mass variations. Adsorption of gas molecules onto the functional layer results in additional mass loading and changes in mechanical damping, leading to a decrease in resonance frequency and modification of the impedance response. The dominant factors affecting resonance include effective mass, membrane stiffness, and damping conditions. A simplified relationship can be expressed as:

$$\frac{\Delta f}{f_{0}}\approx -\frac{1}{2}\frac{\Delta m}{m_{eff}}$$

where Δf is the resonance frequency shift, $f_{0}$ is the initial resonance frequency, Δm is the added mass due to gas adsorption, and $m_{eff}$ is the effective mass of the membrane.

To enable the selectivity and performance of CMUTs towards the target gases, they are functionalized with specific gas-absorbing materials. In our earlier work [22], we explored the application of zeolitic imidazolate framework-8 (ZIF-8), a type of metal–organic framework (MOF), for sensing hydrocarbons. ZIF-8 is characterized by a microporous structure with a pore aperture of approximately 3.4 Å and a cage diameter of about 11.6 Å, which enables selective adsorption of small organic molecules depending on their kinetic diameter. The interaction between VOC molecules and ZIF-8 occurs primarily through physisorption mechanisms [23,24,25], such as van der Waals interactions and weak host–guest interactions within the porous framework. The selected analytes in this study (acetone, isopropanol, ethyl methyl ketone, kerosene, and diesel vapors) have kinetic diameters and molecular structures that allow them to diffuse into or interact with the ZIF-8 pores to varying degrees.

In our work, we found that the humidity of the air in which the gases or vapors are detected has a significant impact on the ZIF-8-modified CMUT characteristics. Therefore, this work aims to further explore earlier found humidity-induced effects by simultaneously introducing humid gases. For this purpose, we used graphene oxide (GOx) as a functional material due to its hydrophilic properties, which make it highly sensitive to changes in water vapor concentration. The GOx-functionalized CMUT-based humidity sensor has quick response and recovery times as was earlier demonstrated by our and some other groups [26]. By employing these functional materials with CMUTs over multiple sensing channels, it is possible to create a versatile sensor capable of detecting multiple gases simultaneously, with improved sensitivity and selectivity compared to conventional methods, combined with unmatched miniaturization and power effectiveness potential.

## 2. Materials and Methods

The functionalization of the capacitive micromachined ultrasonic transducers (CMUTs) was achieved by depositing thin layers of gas-sensitive materials on the device surface. The CMUT devices used in this study were fabricated using standard micromachining techniques for making microelectromechanical systems (MEMS). The devices consist of silicon substrates and membrane structures on top that allow for mass-sensitive oscillations and corresponding measurements.

### 2.1. CMUT Design and Fabrication

The CMUT design parameters were selected based on a trade-off between sensitivity, operating frequency, and fabrication constraints. The operating frequency of approximately 15 MHz was chosen to ensure sufficient sensitivity to small mass changes while maintaining stable operation in atmospheric conditions. Higher frequencies improve sensitivity to small mass changes due to reduced effective mass, but also increase acoustic losses in air and reduce quality factor. Lower frequencies, while easier to excite, result in reduced sensitivity to surface mass loading. Membrane dimensions, gap height, and layer thickness were optimized to achieve a suitable electromechanical coupling and impedance range compatible with the measurement setup.

The use of multiple cells arranged in an array improves signal robustness and the signal-to-noise ratio, although it does not directly increase intrinsic mass sensitivity. A hexagonal cell layout was selected to increase the fill factor and ensure uniform mechanical behavior across the array.

The CMUTs were fabricated using the sacrificial release method—a process that involves depositing and then removing a sacrificial material layer to create the necessary cavity structure beneath the membrane. This technique allows for good control of the membrane dimensions and cavity height, which are critical for establishing the resonant frequency and overall sensitivity of the device. The general fabrication steps are as follows:

1. Deposition of the sacrificial layer onto a silicon wafer.
2. Patterning the sacrificial layer using photolithography to define the areas where the cavities would be formed.
3. Deposition of the membrane material over the patterned sacrificial layer.
4. Etching access holes, followed by the removal of the sacrificial layer using a selective etching process, creating the sub-membrane cavities.
5. Deposition of the sealing layer to vacuum-seal the cavities and access holes and complete the CMUT structure.

**Figure 1:**
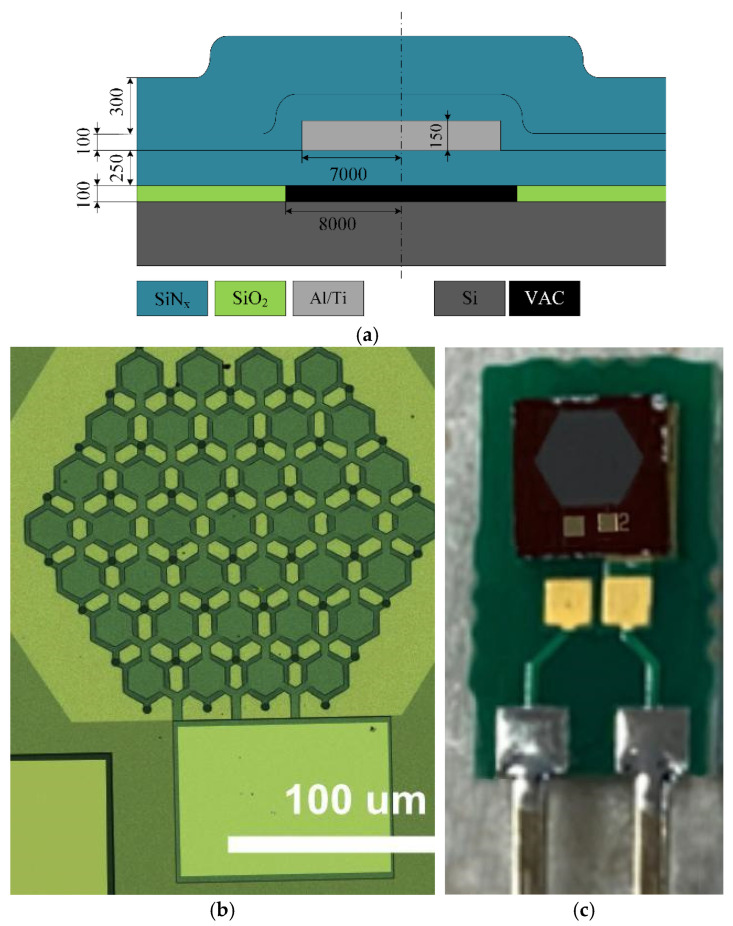
CMUT structure: (**a**) the designed cell cross-section view with dimensions shown in nanometers; (**b**) a micrograph of a fabricated 37-cell device; (**c**) a 2977-cell device die assembled on a PCB.

The sacrificial release method offers several advantages, including lower fabrication costs and the ability to create smaller, more compact devices. The critical dimensions, materials, and overall design of the device are shown in Figure 1a. Devices were designed for 15 MHz resonance in air. For technology testing purposes, devices consisting of 37 cells, with less than 0.2 mm diameter and with adjacent space occupied by contact pads were fabricated. The optical microscopy image of the fabricated 37-cell device is shown in Figure 1b. Along with the 37-cell devices, the full-size devices of 2977 cells with slightly less than 1 mm diameter were fabricated. The number of cells in the array was selected to achieve a measurable electrical impedance while maintaining individual cell sensitivity. Increasing the number of cells improves signal strength due to parallel electrical contribution but also increases parasitic capacitance. The chosen configuration represents a balance between signal amplitude and device sensitivity. A sample device is shown in Figure 1c assembled on the printed circuit board. The overall dimensions of the fabricated die were less than 2 × 2 mm. The (a) part of Figure 1 shows the cross-section of the single CMUT cell design. The device was fabricated over a highly doped (resistivity < 0.01 Ω·cm) silicon substrate with a nominally deposited 100 nm of thermal silicon dioxide. The cavities were etched down to the oxide, and then a 100 nm thick sacrificial layer of chromium was deposited and patterned. The critical thickness of 250 nm of low-stress PECVD silicon nitride was deposited to form the membrane layer, over which the 200 nm thick Ti/Al/Ti metal stack was deposited and patterned to establish the interconnected network of top electrodes. The top electrodes were passivated by depositing an additional 100 nm thick PECVD silicon nitride film. Then the etching access holes were opened, and the sacrificial film was wet-etched, followed by a freeze-drying process. The former steps formed the suspended CMUT membranes. Cavities were sealed and devices finished by depositing another 300 nm silicon nitride film. Lastly, the contact pads for the ground and top contact pads were opened by the lithography of the silicon nitride layer. A fabricated device is shown in the optical micrograph in part b of Figure 1. After dicing, the devices were assembled on a printed circuit board (PCB), using wire bonding. A fully assembled device is shown in Figure 1c. A more detailed device production process description can be found in our previous work [27].

In our previous work, we designed square membranes. They are easier to design and manufacture. This time, hexagonal cells, which have a larger effective working area (corners of square cell have negligible displacement) and therefore better signal, were implemented. The 37-cell device was manufactured as a proof of concept to test the geometry and to demonstrate it. All the functionalization and measurement work was carried out on 2977-cell devices. The larger arrays of CMUT cells result in better signal and therefore better sensitivity.

### 2.2. Preparation of Functional Layers

#### Zeolitic Imidazolate Framework-8 (ZIF-8) Synthesis

The ZIF-8 was synthesized by dissolving zinc nitrate hexahydrate and 2-methylimidazole in separate methanol solutions, which were then combined under continuous stirring at room temperature. To improve adhesion of the ZIF-8 to the CMUT surface, the synthesized ZIF-8 was mixed with the photoresist AZ1215HS (AZ). AZ was diluted 50x with methanol and then combined with ZIF-8 solution in a 1:1 ratio as described in our previous work [22]. The resulting ZIF-8 and photoresist mixture was drop-coated onto the CMUT surface to create a uniform layer. The devices were then allowed to dry under ambient conditions to ensure a stable coating [22].

### 2.3. Graphene Oxide (GOx) Preparation

For humidity sensing, 2 mg/mL aqueous GOx suspension (Merck KGaA, Darmstadt, Germany) was diluted to 0.2 mg/mL and spin-coated onto the CMUT surface at a speed of 2000 rpm for 60 s, forming a thin, uniform layer. No additional baking or curing steps were applied, as the spin-coating process alone was sufficient to secure the GOx layer on the CMUT surface.

### 2.4. CMUT Functionalization Process

The CMUT devices were functionalized by coating the prepared compounds of ZIF-8, GOx directly onto the CMUT surface. ZIF-8 was applied via drop-coating, while GOx was deposited using spin-coating. These methods were chosen to ensure uniform thin films, essential for maintaining consistent sensor performance across the CMUT array. In all cases we used the photosensitive polymer (photoresist) AZ1512HS (Merck KGaA, Darmstadt, Germany) as a matrix for ZIF-8 (as described previously) and GOx particles (the 0.2 mg/mL aqueous solution was mixed at a 1:1 ratio). After deposition, the devices underwent UV exposure followed by curing in the oven to stabilize the polymer. The AZ-based functional layer thickness was 300 nm for spin-coating and 800 nm for drop-coating. The detailed recipe of compound preparation and deposition is proprietary and will not be published in this article. Functionalized devices were kept at a temperature of 20 °C, 40–50% humidity, and in complete darkness when not used for measurements.

### 2.5. Testing

**Figure 2:**
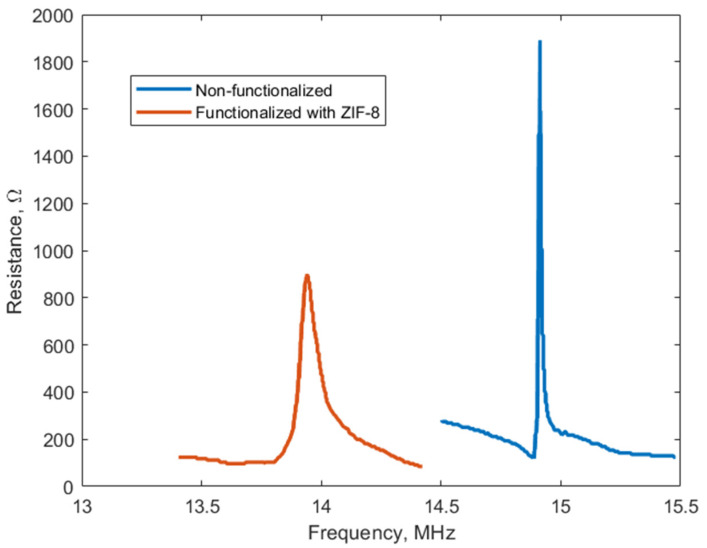
Frequency spectra of the CMUT electromechanical impedance before and after functionalization with ZIF-8. The measurements were performed with a DC bias of 50 V applied to the CMUT device.

After the fabrication, dicing, wire bonding, initial unmodified surface testing and printed circuit board assembly, devices were tested in real-time gas exposure experiments. The resonant frequency and real part of the impedance (resistance) values were measured using a network analyzer, which was complemented with a real-time data acquisition software running on the personal computer for real-time monitoring of the CMUT’s response to gas adsorption. The electromechanical impedance of the CMUT was measured by tracking the electrical input impedance of the device in the vicinity of its resonance frequency, as described in previous studies. Figure 2 depicts the spectra of the real part of the impedance (resistance) measured before and after functionalization of the device with ZIF-8. It is obvious that adding the functional film to the CMUT structure has damping and mass increase effects over the mechanical part of a device, since the resonance quality and local maximum of the resistance readings becomes decreased, and the resonance frequency is decreased too. Both measurements were taken in the ambient air, so there was no to only negligible interaction of the functional film with the gaseous medium present around the sensor, since we have previously demonstrated that N_2_ and O_2_ present in the air do not interact with ZIF-8 and trace contaminants are found at diminishingly low concentrations [22]. Humidity would affect the sensors as demonstrated in the later results, but here the effect of functional layer deposition is demonstrated. The GOx-functionalized devices demonstrated similar results.

In addition to the frequency shift, a reduction in the Q-factor is observed, indicating increased mechanical damping due to mass loading introduced by the functional layer. This effect is likely related to viscoelastic losses within the functional layer and additional energy dissipation mechanisms introduced by the coating.

**Figure 3:**
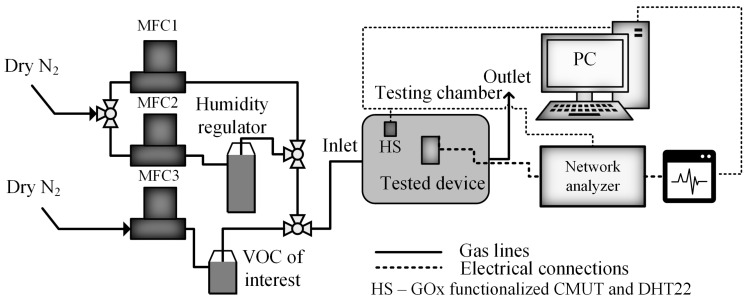
Gas and vapor control system for sensor testing.

The gas flow was controlled using a custom-built system equipped with mass flow controllers, enabling accurate regulation of gas vapor levels for target gases. The gas mixture control system is shown in Figure 3.

The experiments were conducted in a custom gas chamber, where the functionalized CMUTs were exposed to controlled environments. The network analyzer was used to record changes in the resonant frequency and impedance, and PC software was used for the real-time tracking of two parameters of the impedance spectra (illustrated in Figure 2): the resonance frequency, which was read on the frequency axis at the local maximum of the spectra, and the maximum of the resistance reading. This setup allowed for real-time assessment of the sensors’ sensitivity, response time, and recovery time, without the need for additional calibration curves.

Gases or vapors of interest were flowed through the testing chamber for 10 min, then switched to purging with nitrogen gas for 10 min. This cycle was repeated at least 5 times in each experiment. Each experiment was repeated at least 10 times with different CMUT devices to test the repeatability and reproducibility of the sensor response for each material and sensor. The system was designed and operated in a continuous-flow, non-sealed configuration with an open outlet. Under these conditions, gas exchange occurs freely within the test chamber, preventing pressure buildup inside the chamber and helping avoid any pressure effects on the sensor’s readings. All the measurements were conducted at 20 °C.

The gas of interest was transported to the measurement chamber, in which the functionalized CMUT device was placed in the middle of the gas chamber with as little gas flow obstruction as possible. The gas and vapor flow was regulated through the mass flow controller MFC3, according to Figure 3. Controlled humidity was achieved by adding compressed nitrogen that passed through the humidity regulator and the mass flow controllers MFC1 and MFC2 and the VOC injected via MFC3, by bubbling dry N_2_ through a container with a liquid VOC species. A commercial humidity sensor DHT22 and our GOx-functionalized humidity sensors were used to monitor the humidity. The final flow rate into the testing chamber was 100 sccm. While the gas/vapor mixture arrived in the sensor chamber, the functional film of CMUT interacted with the vapor by adsorption/desorption mechanisms, thus changing the mechanical load of the CMUT structure.

### 2.6. Real-Time Humidity Measurement

**Figure 4:**
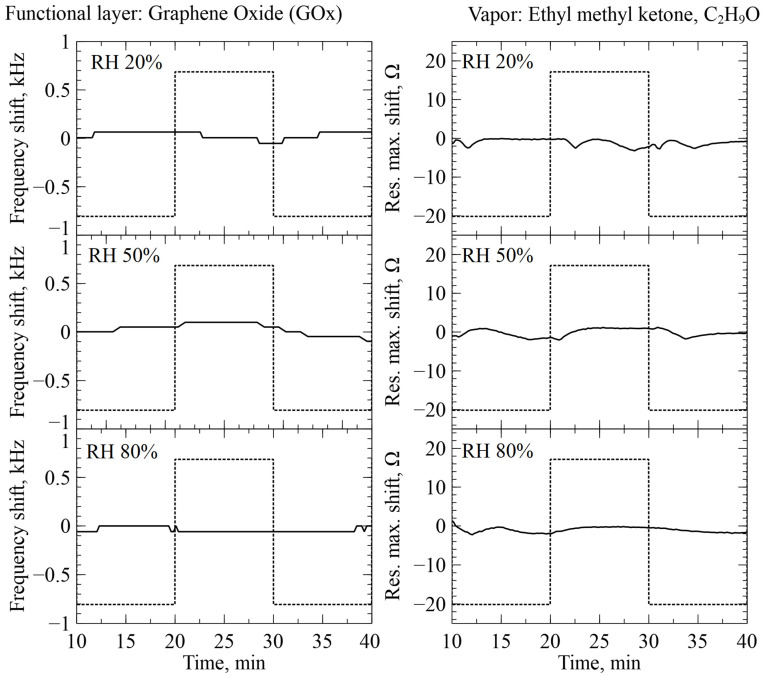
Real-time readings of GOx-functionalized CMUTs during the introduction of EMK at different relative humidity levels. Dotted graphs show the period of EMK vapor introduction into the testing chamber. The axes “Res.max.shift” represent resistance maximum shift.

**Figure 5:**
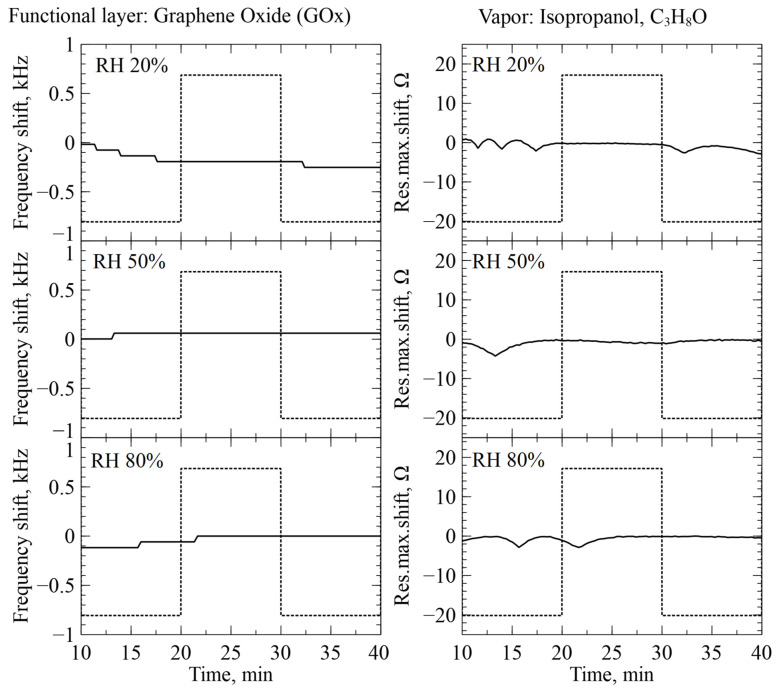
Real-time readings of GOx-functionalized CMUTs during the introduction of isopropanol at different relative humidity levels. Dotted graphs show the period of EMK vapor introduction into the testing chamber. The axes “Res.max.shift” represent resistance maximum shift.

For increased reliability of our experiments and real-time humidity control, we used CMUTs functionalized with graphene oxide (GOx). The technique of functionalization and response of GOx-functionalized sensors is described in our previous publications [22,26,27]. To estimate any possible interaction between the GOx-functionalized CMUTs and the selected organic molecules, ethyl methyl ketone (EMK) and isopropanol were introduced to the measurement chamber under different humidity conditions for the functionalized sensors [28]. The results of the real-time tests are illustrated in Figure 4 and Figure 5. When the GOx-functionalized sensors were adjusted for humidity, no significant correlation between the introduction of the organic vapors and sensor readings were observed. The registered fluctuation of the readings can be estimated as non-significant, on the level of noise and having an erratic character, most likely related to the transitional kinetic processes of gas switching. This might indicate that there is very little to no interaction between GOx and the two selected organic compounds. Thus, we considered our GOx-functionalized CMUT sensors as reliable humidity sensors without any need for correction for all other experiments. This along with DHT22 allowed for reliable manual humidity control in the subsequent experiments.

## 3. Results

The electromechanical impedance of CMUT immediately responds to the changing physical state of the applied functional layers, producing shifts in the resonant frequency and maximum resistance readings. According to the well-known lumped element model of CMUT structure [29,30,31,32], the resonance frequency shift can be attributed to the changing mass and overall mechanical stiffness of the functionalized CMUT membrane, and the change in the resonance spectrum maximum value can be attributed to the changing flexibility or other loss mechanisms of the functional layer.

**Figure 6:**
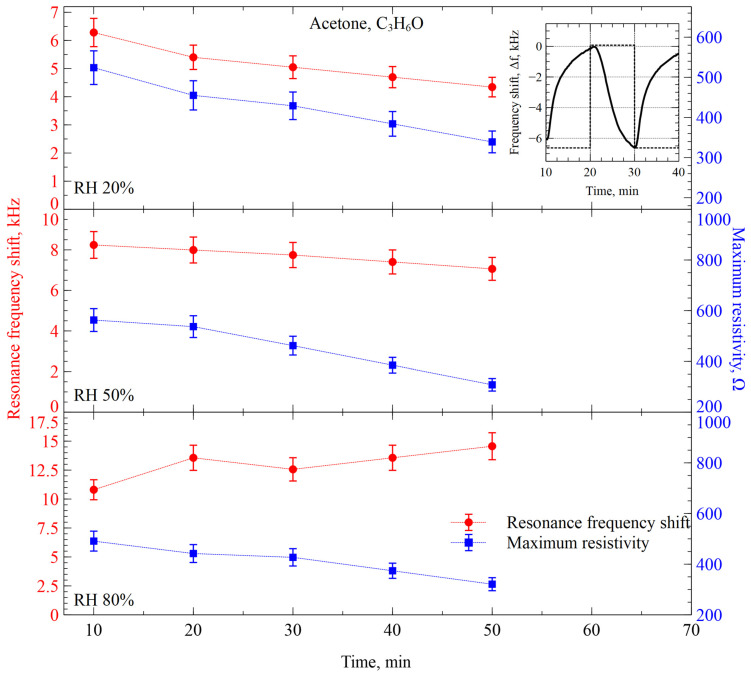
Changes in resonance frequency and resistance maximum shift in the CMUT-based gas sensor functionalized with ZIF-8 interacting with acetone vapor over time at different relative humidity levels. The inset shows a single-step response of the sensor to the switching between acetone vapor and humidity-compensated nitrogen gas. The dashed line indicates switching points.

Experiments were started by measuring the interaction of ZIF-8-functionalized CMUTs with acetone (C_3_H_6_O). The molar mass of an acetone molecule is 58.08 g/mol, which is the lowest-molecular-mass organic molecule explored in this work. Figure 6 depicts the readings of the resonance frequency and resistance maximum shifts when a ZIF-8-functionalized CMUT interacts with acetone vapor at three different relative humidity conditions. While the inset in the figure exemplifies the real-time pattern of the resonance frequency shift, the main graph shows the values of the resonance frequency shift and maximum resistance at each measurement cycle after N_2_ gas purge on the same device. Both parameters exhibit good stability and signal-to-noise ratio, showing approximately 6 kHz resonance frequency shift and 500 Ω resistance maximum shift, which are 0.04% and 25% of the nominal values of the resonance frequency and resistance maximum, respectively. The error bars indicate the scatter of the measured readings over 10 different devices.

Interaction with acetone induces a negative resonance frequency shift (decrease from the initial frequency) of the functionalized CMUTs and a similar negative shift (decrease from the initial resistance) of the resistance maximum. While the interaction is almost fully reversible within the single cycle of an experiment, since the impedance reading partially returns toward its initial value, indicating reversible adsorption, a residual drift is observed, likely due to the slow desorption or structural changes in the functional layer. This observed drift is largely reversible over extended recovery periods. However, slower irreversible components may occur due to dielectric charging effects. When the chamber gets purged with nitrogen, some drift over repeating interactions is apparent. Completely steady state or saturation of the interaction was not achieved over one hour of experiment. Additionally, we can observe that there is a significant correlation between the interaction of resistance maximum shift and the relative humidity of the gas/vapor mixture. While in considerably dry conditions of 20% RH the resistance maximum shifts are approximately 6 kHz and 500 Ω, increasing humidity to 50% causes the resonance frequency shift to increase to approximately 8 kHz. While the humidity increases to 80%, the resonance frequency shift reaches 10–15 kHz. Also, within humid conditions, there is no longer an obvious trend in the frequency shift readings. At the same time, we see only minor influence or no influence at all to the resistance readings, since the resistance maximum shift remains generally the same, with a slight negative trend. We interpret these observations as meaning that acetone is intensively absorbed by the functional film, increasing the moving mass, and the presence of the water vapor is seemingly to promote the absorption intensity. However, the adsorption/absorption layer thickness is difficult to estimate as the full mechanism and diffusion rates are not known. At the same time, increasing humidity does not cause any significant changes in the mechanical damping of the CMUT structure, since the shift in the resistance maximum value stays virtually unchanged.

**Figure 7:**
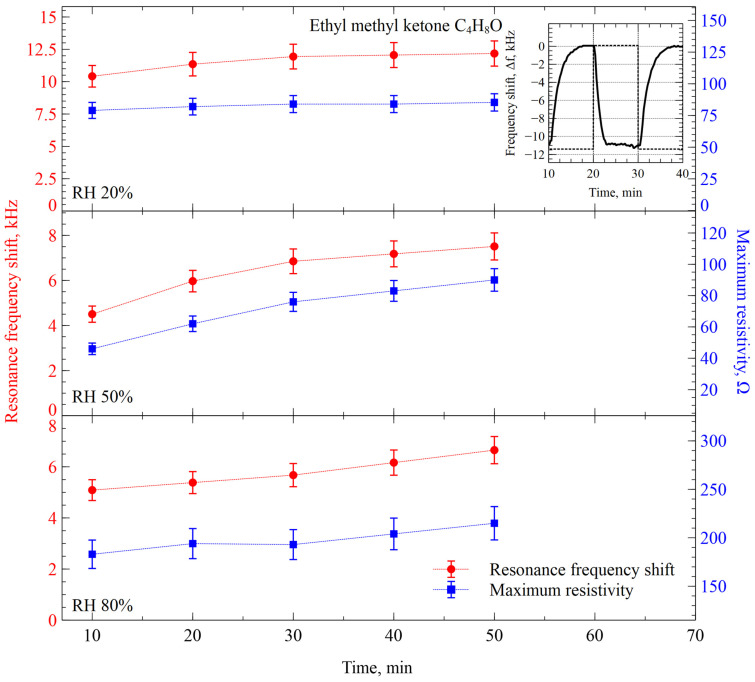
Resonance frequency and resistance maximum shifts during the interaction of CMUTs functionalized with ZIF-8 with EMK vapor over time at different relative humidity levels. The inset shows a single-step response of the sensor to the switching between ethyl methyl ketone vapor and humidity-compensated nitrogen gas.

The next experimental step was done with EMK (C_4_H_8_O), whose molecules are more complex than acetone in terms of number of carbons and weight, with a molecular mass of 72.19 g/mol. The experiment was performed by preserving the same conditions as before. In Figure 7 we show the response of the CMUT resonance frequency and resistance shifts to the interaction between EMK and the functional film, containing ZIF-8 and AZ1512HS.

It can be easily detected that in low humidity conditions of 20% RH, the interaction with EMK is fully reversible, with only minor signs of the positive trends, i.e., increasing both shifts over the one-hour experiment. Also, the resonance frequency shift is higher than the one for acetone: 10–12 kHz instead of 6 kHz for the same conditions. At the same time, the resistance shift is much smaller, compared to the acetone experiment, producing less than 100 Ω shift. Upon increase in the humidity to 50%, the positive trends in both measurement channels become more pronounced, but the amplitudes of the interaction signals decrease. While humidity is increased to 80%, it causes an increase in the average readings of the resistance channel, but the frequency shift channel shows precisely the same response as with 50% humidity. Both types of reading preserve the same well-expressed positive trend.

Similarly, as for the acetone experiment, we can conclude here that there is a significant relationship between the humidity of the gas/vapor mixture and the response of our gas sensing system. Also, the increased resonance frequency shift response to the increased molecular mass is easily explainable; however, this explanation does not hold fully, because increasing humidity discards the consistency. This inconsistency can be explained by the complex interaction kinetics, attributable to ZIF-8 film surface properties, with some parts of ZIF-8 layers saturating and desaturating faster. This effect has been observed before [22].

**Figure 8:**
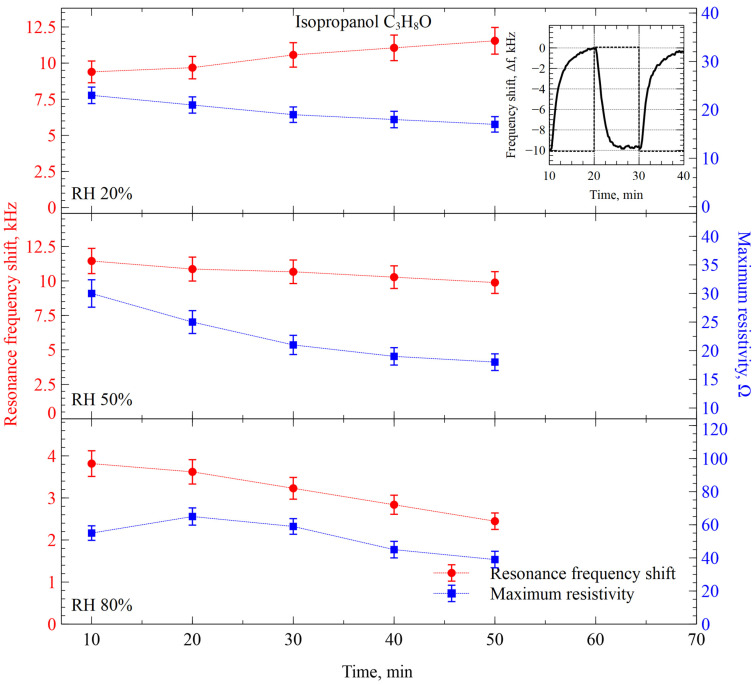
Resonance frequency and resistance maximum shifts of the CMUT-based gas sensor functionalized with ZIF-8 interacting with IPA at different relative humidity levels. The inset shows a single-step response of the sensor to the changes between IPA vapor and humidity-compensated nitrogen gas.

In our next experimental step, we observed a similar interaction between the isopropanol (IPA, C_3_H_8_O) with molecular mass of 60.1 g/mol and CMUTs functionalized with the same film, containing ZIF-8. The isopropanol molecule is only slightly more complex than acetone, with minor differences in molecular mass, while both are lighter and simpler than the EMK molecule. Therefore, it was expected to observe vapor/sensor interactions that resemble previous cases of the acetone. Time diagrams depicting the evolutions of CMUT’s resonance frequency and resistance maximum shifts are shown in Figure 8. 

As expected, the IPA-induced evolutions of both CMUT impedance parameters fell somewhere in between acetone and EMK. While a positive trend of the resonance frequency shift was observed within the dry environment (20% RH), increasing humidity to 50% induced the similarity of the readings to the acetone-like pattern with increased resonance frequency shift and noticeable negative trends over time. Further, when the relative humidity was increased to 80%, the IPA-induced evolutions of CMUT impedance parameters resembled those of EMK, since the average amplitude of the resonance frequency shift decreased considerably, while the response of the resistance maximum shift remained generally the same.

**Figure 9:**
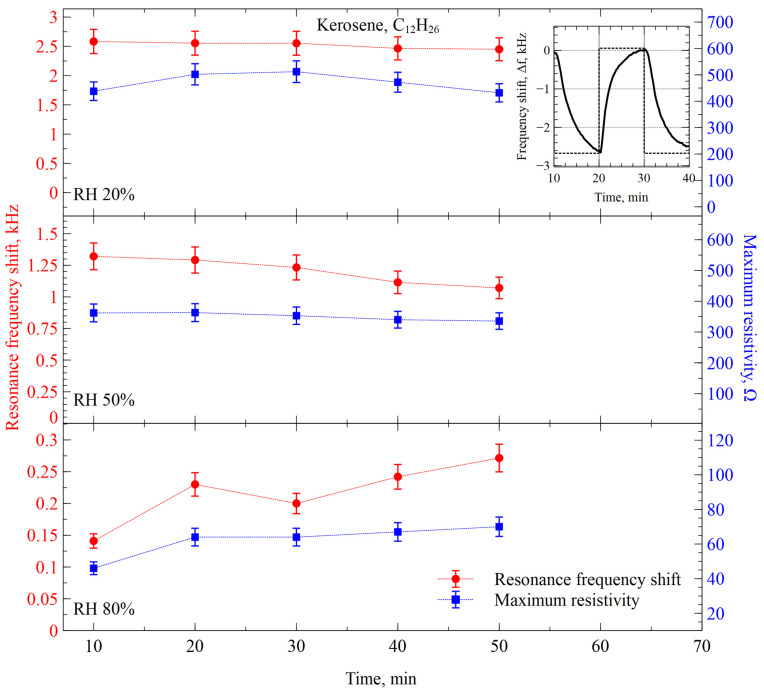
Resonance frequency and resistance maximum shifts of the CMUT-based gas sensor functionalized with ZIF-8 interacting with kerosene vapor at different relative humidity levels. The inset shows a single-step response of the sensor to the changes between kerosene vapor and humidity-compensated nitrogen gas.

Kerosene vapor was used to test our sensing system further. Kerosene is a complex mixture of hydrocarbons, primarily in the C_10_ to C_16_ range. It does not have a single chemical formula like pure compounds in our previous experiments do, but it is generally described as a hydrocarbon mixture containing mostly alkanes and cycloalkanes ranging from C_10_H_22_ to C_16_H_34_. Its typical molecular mass is between 140 and 200 g/mol. While consistency of the sensing response with molecules with larger mass was expected, the actual data of the experiment showed the opposite. The results are illustrated in Figure 9. The interaction of kerosene with our sensing system exhibited much lower shifts in the resonance frequency, if compared to any of the previous substances (acetone, EMK and IPA). However, the resistance maximum shift was of similar amplitude to the case with acetone, ranging from 300 to 500 Ω, in the case of 20% and 50% RH values. While the humidity was increased to 80%, the resistance shift amplitude fell to less than 100 Ω. The trends over a one-hour period were negligible for both parameters in all cases, indicating full reversibility of the functional film over the explored conditions.

To explore further the response of our sensing system to the mixed alkanes, we tested it with diesel vapors. Like kerosene, diesel is also a complex mixture of hydrocarbons, primarily in the C_10_ to C_20_ range, i.e., the hydrocarbons ranging among alkanes (paraffins), cycloalkanes (naphthenes), and some aromatic hydrocarbons. The range of molecule complexity in the diesel mixture is not only wider but also on the heavier side. Like kerosene, diesel does not have a single chemical formula (ranging between C_10_H_20_ and C_20_H_42_), but its molecular mass is typically estimated to be between 170 and 250 g/mol.

**Figure 10:**
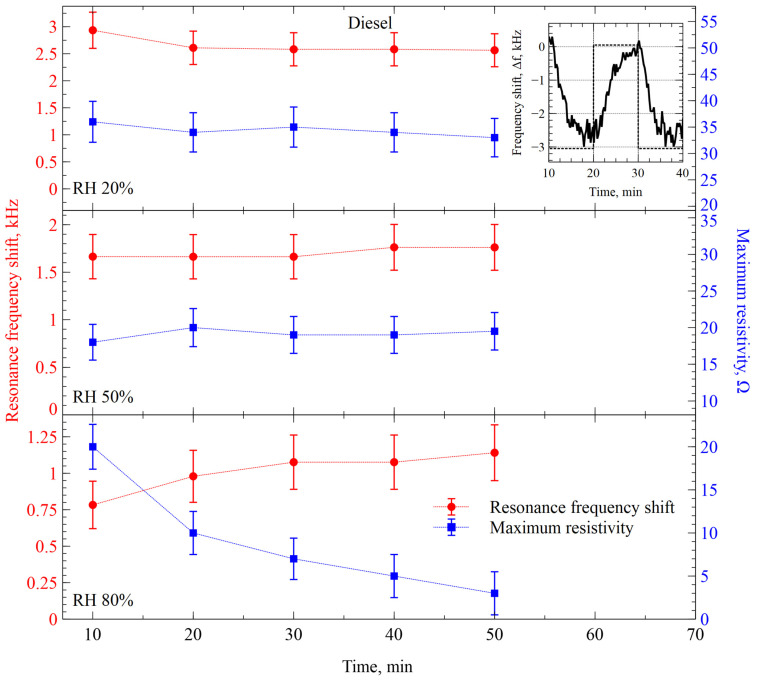
Resonance frequency and resistance maximum shifts of CMUT-based gas sensor functionalized with ZIF-8 interacting with diesel vapor at different relative humidity levels. The inset shows a single-step response of the sensor to the changes between diesel vapor and humidity-compensated nitrogen gas.

Readings of our sensing system in the case of the tests with diesel vapors are illustrated in Figure 10. While the resonance frequency shifts are very similar to those of kerosene in all cases of relative humidity, the resistance maximum shifts are generally lower. As for the one-hour trend, the parameters exhibited different dynamics in the case of 80% RH: the resonance frequency shift increased, and the resistance maximum shift decreased from 20 Ω to less than 5 Ω. Again, we are explaining it by the unexplored interaction kinetics and some possible anomalies in the functional films.

The interaction of ZIF-8 with diesel vapor demonstrated similar trends as with kerosene (Figure 9). Both the frequency shift and resistance maximum shift change was positive with the introduction of diesel vapor into the test chamber. However, diesel vapor frequency shift values were much more unstable over time compared to kerosene readings, even though the measured frequency shift between the gas of interest and nitrogen were in the range of single-digit kHz for both diesel and kerosene frequency readings. Additionally, whereas kerosene amplitude readings between nitrogen and kerosene changed by 100s of Ωs, the diesel amplitude difference was only in 10s of Ωs.

**Figure 11:**
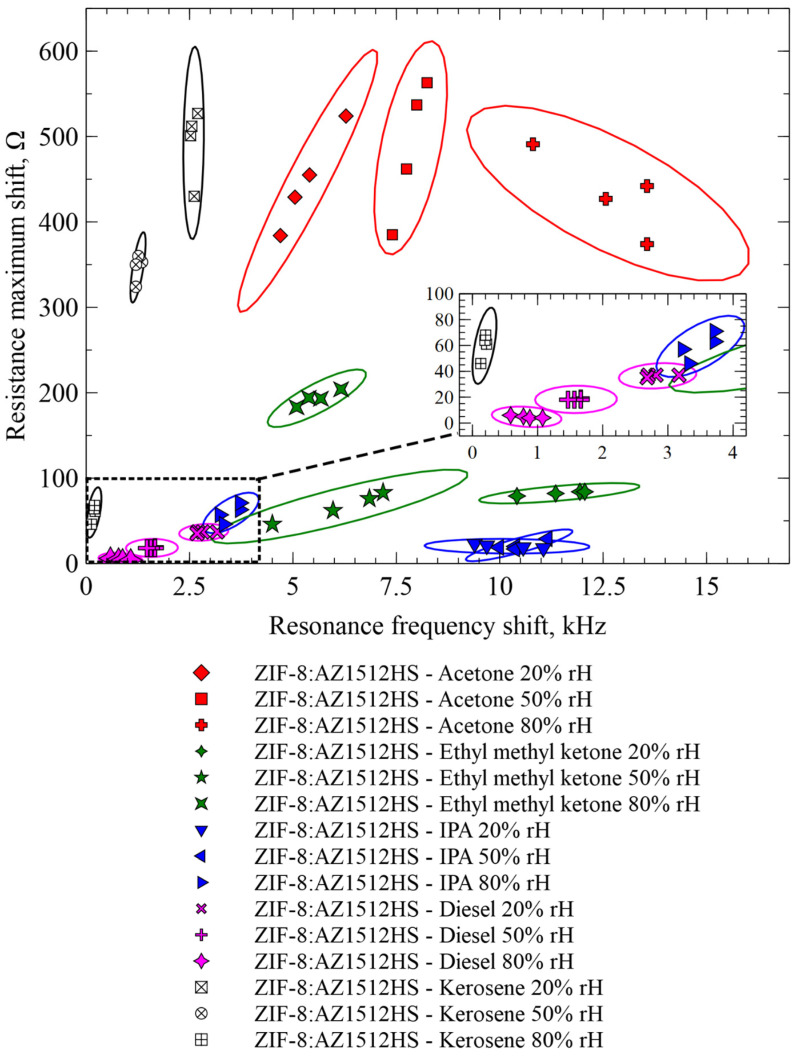
Readings of the ZIF-8 functionalized CMUTs summarized over all experiments. The inset shows a zoomed version of the graph area marked with the dashed rectangle. Each measurement condition was repeated four times on a different chip to ensure repeatability and to account for variability arising from gas adsorption/desorption dynamics, environmental fluctuations, and measurement noise.

To summarize the results of our experiments, Figure 11 illustrates the responses of our sensing system to all the vapors that were used in this work. Each measurement condition was repeated four times to ensure repeatability and to account for device-to-device variability. Sensor readings are plotted on a single chart with the resonance frequency shift on the horizontal axis and the resistance maximum shift on the vertical axis. While the demonstrated technique is relatively weak in descriptivity as compared to spectroscopic techniques, such as FTIR, it is obvious that the tracking of both impedance parameters allows for distinction local to the experimental conditions (under certain conditions, such as known humidity, gas concentration and identified VOC species) between organic vapors if they are introduced in a controlled manner as we did in our experiment.

**Figure 12:**
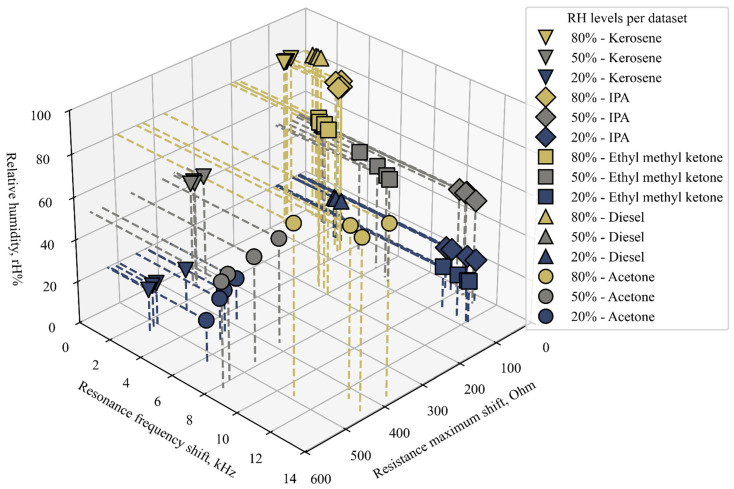
Readings of the ZIF-8 functionalized CMUTs summarized over all experiments with humidity having a separate dedicated axis. The dashed lines show data point projections onto relevant planes.

To better demonstrate the influence of humidity on system readings, a 3D plot where the third axis is humidity was produced (Figure 12). This plot is a variation of Figure 11. Even though the majority of data points from any gas species do not shift onto another species’ values when relative humidity is adjusted, a lack of discernibility in such cases can be expected. For example, IPA, measured at 80% rH, produces resistance maximum shift and resonance frequency values close to 20% diesel vapor data (the IPA data points are located directly above the diesel points). Similarly, other materials, for which the sensor has relatively low sensitivity (other values of diesel vapor; kerosene at 80%), are also close to overlapping. This underlines the fact that the sensing system can discern species only under certain conditions, namely, known humidity. Gas concentration also affects the sensor response and may lead to overlaps between different VOCs. Mapping response trajectories over a wider concentration range in the resonance frequency and resistance maximum shift space would help clarify the extent of such overlap, particularly for ZIF-8 and other functional materials that may exhibit coating-specific concentration-dependent patterns. However, performing extensive concentration sweeps without first understanding humidity-induced response shifts may result in misleading calibration trends. Because humidity influences both mass loading and damping-related parameters, it alters not only the response magnitude but also the trajectory of data points in the impedance space. Therefore, characterizing humidity-dependent behavior is a prerequisite for reliable concentration mapping.

## 4. Conclusions

While our observations are local to the experimental conditions described, we still can conclude that ZIF-8 is a suitable functional material for the detection of a wide range of hydrocarbon-based VOCs. By simultaneously monitoring resonance frequency shift and resistance maximum shift, we were able to analyze the multidimensional electromechanical response of the sensor under controlled humidity conditions. As can be seen from Figure 11, despite the drift of the readings, which was present in some cases, and also despite the drastic range of humidity conditions, the measured points presented on the resonance and resistance axes tend to group in a specific region of the coordinate system. We also demonstrated that relative humidity had a significant impact on the measured readings, even though in this case it mostly did not affect molecular species discernability.

A key outcome of this study is the clear demonstration that relative humidity significantly influences both impedance-derived parameters. Humidity alters the position and trajectory of response points in the resonance frequency–resistance space, highlighting the importance of environmental control and interpretation in gravimetric sensing systems.

Considering that this is a gravimetric sensor and that CMUT-based sensors have already been shown to be concentration-sensitive, further research should focus on sensor reading changes when the selected VOC gas and vapor concentrations vary and on how that affects VOC discernibility. Considering that CMUT devices can be produced in large quantities in parallel (hundreds of devices on a single typical silicon wafer), a system composed of only CMUT-based sensors with different sensing functional material layers (e.g., zeolites with different pore sizes) could in principle allow for more selective VOC absorption and differing concentration change lines in sensor resonance shift and resistance maximum shift space for different materials, eliminating any problems with discernibility, just as GOx as a functional material allowed for much needed humidity monitoring.

Another observation is that the interaction between functional material and targeted VOCs is complex and needs more thorough investigation to reveal the actual physical and chemical processes that are responsible for these results. While the interactions were mostly reversible, some cases exhibited obvious non-reversible trends, which we speculate relate to the gradual change in the properties of thicker parts of the functional films, most likely in the polymer matrix material, which might potentially be susceptible to chemical reactions with the vapors of acetone and EMK.

The GOx-functionalized CMUTs were instrumental in this research, and we have ensured that they do not show significant interaction with the target vapors, even if they use the same matrix material as the ZIF-8-functionalized devices. Therefore, they were proven to be suitable for reliable in situ and real-time control and monitoring of humidity.

This work contributes to the methodological development of impedance-based MEMS gas sensing by demonstrating that environmental parameter control and multidimensional response analysis should precede full concentration calibration to ensure reliable interpretation.

## Figures and Tables

**Table 1:** Summary of different types of gas sensing technologies.

Technology	Advantages	Disadvantages
NDIR [4,5]	High selectivity; robust	Bulky; expensive; limited to gases with IR absorption
FTIR [6,7]	High selectivity; high sensitivity	Expensive; bulky; limited to gases with IR absorption
Chemiresistive [8,9]	Low cost; simple design; high sensitivity	Susceptible to environmental changes; limited selectivity; high energy usage
MOS [10,11]	Good sensitivity; low energy usage	Cross-sensitivity issues; drift over time
Electrochemical [8,12] (overall)	High selectivity; cheap	Short lifespan; drift over time
Optical [4,5,6,7] (overall)	Fast response; no chemical reaction present	Complex design; higher cost

## Data Availability

The datasets presented in this article are not readily available because the data is part of an ongoing study. Requests to access the datasets should be directed to the corresponding author.
